# Pulmonary Hypertension: Epidemiology in Different CKD Stages and Its Association with Cardiovascular Morbidity

**DOI:** 10.1371/journal.pone.0114392

**Published:** 2014-12-19

**Authors:** Zhilian Li, Xinling Liang, Shuangxin Liu, Zhiming Ye, Yuanhan Chen, Wenjian Wang, Ruizhao Li, Lixia Xu, Zhonglin Feng, Wei Shi

**Affiliations:** 1 Department of Nephrology, Guangdong General Hospital, Guangdong Academy of Medical Sciences, Guangzhou, China; 2 Southern Medical University, Guangzhou, China; Indiana University, United States of America

## Abstract

**Background:**

Pulmonary hypertension (PH) was recently recognized as a common complication of end-stage renal disease (ESRD) that causes an increased risk of mortality. Epidemiological data for this disorder in earlier stages of chronic kidney disease (CKD) and its association with cardiovascular (CV) morbidity are scarce.

**Methods:**

We retrospectively analyzed 2,351 Chinese CKD patients with complete clinical records and echocardiography data between Jan 2008 and May 2012. The patients were divided into the following 6 groups: CKD Stages 1–4; Stage 5 for those not on or initiated on hemodialysis for <3 months; and Stage 5D for the patients undergoing hemodialysis for ≥3 months. The prevalence of PH and CV morbidity was investigated, and their association was evaluated with a logistic regression model.

**Results:**

PH was detected in 426 patients (18.1%). Mild, moderate and severe PH was diagnosed in 12.1%, 4.9% and 1.1% of the patients, respectively. Severe PH was detected in CKD Stages 5 and 5D. CV morbidity was found in 645 patients (27.4%). Compared with the non-PH group, the PH group had a higher risk for cardiac disease but not for cerebrovascular disease risk. PH severity was associated with cardiac morbidity risk [odds ratio (95% CI) for mild PH: 1.79 (1.30–2.47); moderate PH: 2.75 (1.73–4.37); severe PH: 3.90 (1.46–10.42)].

**Conclusions:**

Our study showed for the first time the epidemiology profile of PH across the spectrum of CKD. Mild-to-moderate PH occurs with more frequency in advanced CKD, and severe PH is scarce in non-ESRD CKD. PH in CKD is associated with cardiac but not cerebrovascular disease, with increasing cardiac morbidity seen with increasing PH severity. Evidence from prospective studies addressing PH in this population is needed to predict cardiac events.

## Introduction

Pulmonary hypertension (PH), a devastating disorder characterized by elevated pulmonary artery pressure (PAP), has been reported as a new entity and an unrecognized threat in a considerable proportion of patients with end stage renal disease (ESRD) [1]–[5]. Based on an echocardiographic diagnosis of PH, the reported prevalence of PH ranges from 9%–39% in individuals with Stage 5 Chronic Kidney Disease (CKD) [6]–[10], from 18.8%–68.8% in hemodialysis patients, and from 0%–42% in patients on peritoneal dialysis therapy [11]–[14]. The exact mechanisms of PH in this population remain poorly understood. PH might be induced and/or aggravated by left ventricular disorders and risk factors typical of CKD, including volume overload, arteriovenous fistula, sleep-disordered breathing, exposure to dialysis membranes, endothelial dysfunction, vascular calcification and stiffening, and severe anemia [2]–[4]. ESRD-related PH, for the first time, was grouped into the 5th subtype (PH with unclear multifactorial mechanisms) of PH by the World Symposium of PH (WSPH) in Dana Point (2008) [15] and then updated in Nice (2013) [16].

Whether PH occurs only in ESRD patients is unknown. It is unclear how and in which stage PH originates. Early diagnosis and early intervention of PH might improve the long-term outcomes [17]. Therefore, it is crucial to investigate the epidemiology of PH before CKD patients progress to ESRD. However, no epidemiological data are available for the earlier stages of CKD. Although a very recent study reported the presence of CKD Stages 3–4 with PH, those patients were selected from a PH registry, and non-PH CKD data were lacking; therefore, the study did not show the actual epidemiology of CKD [18]. Besides, our previous study revealed a prognostic value of PH in predicting cardiovascular (CV) outcomes in maintenance hemodialysis (MHD) patients [19]; whether there is a link between PH and CV disease in other stages of CKD is unknown. The aims of this study were as follows: (1) to investigate the epidemiology and baseline characteristics of PH in different stages of CKD; and (2) to determine the association of PH with CV morbidity in CKD patients.

### Study subjects

The retrospective study was conducted in the Renal Division of the Guangdong General Hospital (GGH) (a kidney disease center in Southern China and the Hemodialysis Quality Control Center of Guangdong Province) in China. Between January 2008 and May 2013, all of the adult in-patients admitted to the GGH Renal Division were screened for eligibility in the GGH Echocardiographic Data System. The inclusion criteria were as follows: (1) 18 years of age or older; (2) diagnosed CKD patients [not including peritoneal dialysis (PD) patients]; patients whose status had changed from PD to HD for longer than 3 months; and (3) complete clinical and echocardiographic data. The exclusion criteria were (1) maintenance PD patients and (2) non-CKD patients. Patients were excluded if they had PH secondary to chronic obstructive pulmonary disease (COPD), collagen vascular disease, a pulmonary embolism or chest wall or parenchymal lung disease, rheumatic heart disease (RHD), congenital heart disease (CHD), acute heart failure (AHF) and portal hypertension [20].

The definition of CKD and the CKD stage were based on the K/DOQI guidelines [21], using the CKD Epidemiology Collaboration (CKD-EPI) formula to estimate the glomerular filtration rate (GFR, ml/min per 1.73 m^2^) [22]. The patients were divided into the following 6 groups: Groups 1–4 for CKD Stages 1–4; Group 5 for those who were in Stage 5 and not on or beginning hemodialysis <3 months; Group 6 (CKD-5D) for maintenance hemodialysis (MHD) patients (hemodialysis ≥3 months).

The Ethics Research Committee at Guangdong General Hospital approved the study and agreed that informed consent was not necessary because of the observational nature of this study. The information of all of the patients was anonymized and de-identified prior to analysis.

### Echocardiographic examination

(1) The detection of PH by echocardiography was as follows: the echocardiographic data were obtained from the GGH Echocardiographic Data System. For the patients with multiple hospitalizations and echocardiographic data, only the first admission (during the study period) was considered in the analysis. M-mode, two-dimensional and tissue Doppler echocardiography were performed for all of the subjects using an Acuson Sequoia C256 (Acuson, Mountain View, CA, USA) echocardiographic imaging system equipped with a 2.0–3.5 MHz transducer. Systolic pulmonary artery pressure (SPAP) was evaluated and calculated using a modified Bernoulli equation: SPAP = 4×(tricuspid systolic jet)^2^ +10 mmHg (estimated right atrial pressure) [23]. A value ≥35 mmHg was defined as PH [20]. The severity of PH was categorized according to the SPAP as follows: mild PH (35–50 mmHg), moderate PH (>50–70 mmHg) and severe PH (≥70 mmHg) [20]. (2) Other echocardiographic findings included the following:

a) Regurgitation valvular disease: moderate to severe regurgitation of the mitral and/or the aortic valve was defined based on the European Association of Echocardiography recommendations for the assessment of valvular regurgitation [24]–[25]; b) Pericardial effusion: the severity of pericardial effusion was characterized as trace to small if the pericardial space was separated by <1 cm in the diastole in any plane. Moderate or severe effusion was defined as a pericardial space separation of >1 cm during the diastole as described previously [26]–[27]; and c) Left ventricular systolic and diastolic dysfunction was defined based on European Society of Cardiology (ESC) Guidelines for the diagnosis of acute and chronic heart failure 2012 and recommendations by the American Society of Echocardiography as well as the European Association of Echocardiography [28]–[29].

### Clinical and laboratory data collection

Information regarding the age, gender, systolic BP (SBP), diastolic BP (DBP), body mass index (BMI), CKD etiology, history of smoking, diabetes, hypertension, and coronary heart disease [including a history of percutaneous coronary intervention (PCI) or coronary artery bypass grafting (CABG)], other CV morbidities as well as laboratory parameters were recorded. The laboratory parameters included a 24-hour collection of proteinuria (not including CKD 5D patients because patients in this stage were usually anuria), hemoglobin, serum albumin, serum uric acid, cholesterol, triglyceride, high-density lipoprotein (HDL) and low-density lipoprotein (LDL).

### Evaluation of CV morbidity

CV morbidity was evaluated according to records from the computerized hospital database, which included cardiac [AHF, angina, acute myocardial infarction, sudden cardiac arrest (or sudden death), arrhythmia requiring hospitalization], cerebrovascular (transient ischemic attack, thromboembolic or hemorrhagic stroke) and peripheral vascular diseases [30]. AHF was defined as either new-onset HF or as the decompensation of chronic HF with symptoms sufficient to warrant hospitalization. The diagnosis of AHF was based on the European Society of Cardiology Criteria [28].

### Statistical Analysis

The categorical data were summarized as numbers and percentages, and differences were tested with the χ^2^ test. The continuous variables were summarized as medians and interquartile ranges or means and SDs, and differences were tested with Student's *t* test for the normally distributed variables and with the Wilcoxon rank sum test for the non-normally distributed variables. The clinical variables associated with PH or CV morbidity were evaluated using a stepwise forward logistic regression analysis. Differences were considered statistically significant at the two-sided P<0.05 level. The statistical analyses were performed using the SPSS statistical package (Version 16.0, Chicago, IL, USA).

## Results

**Figure 1:**
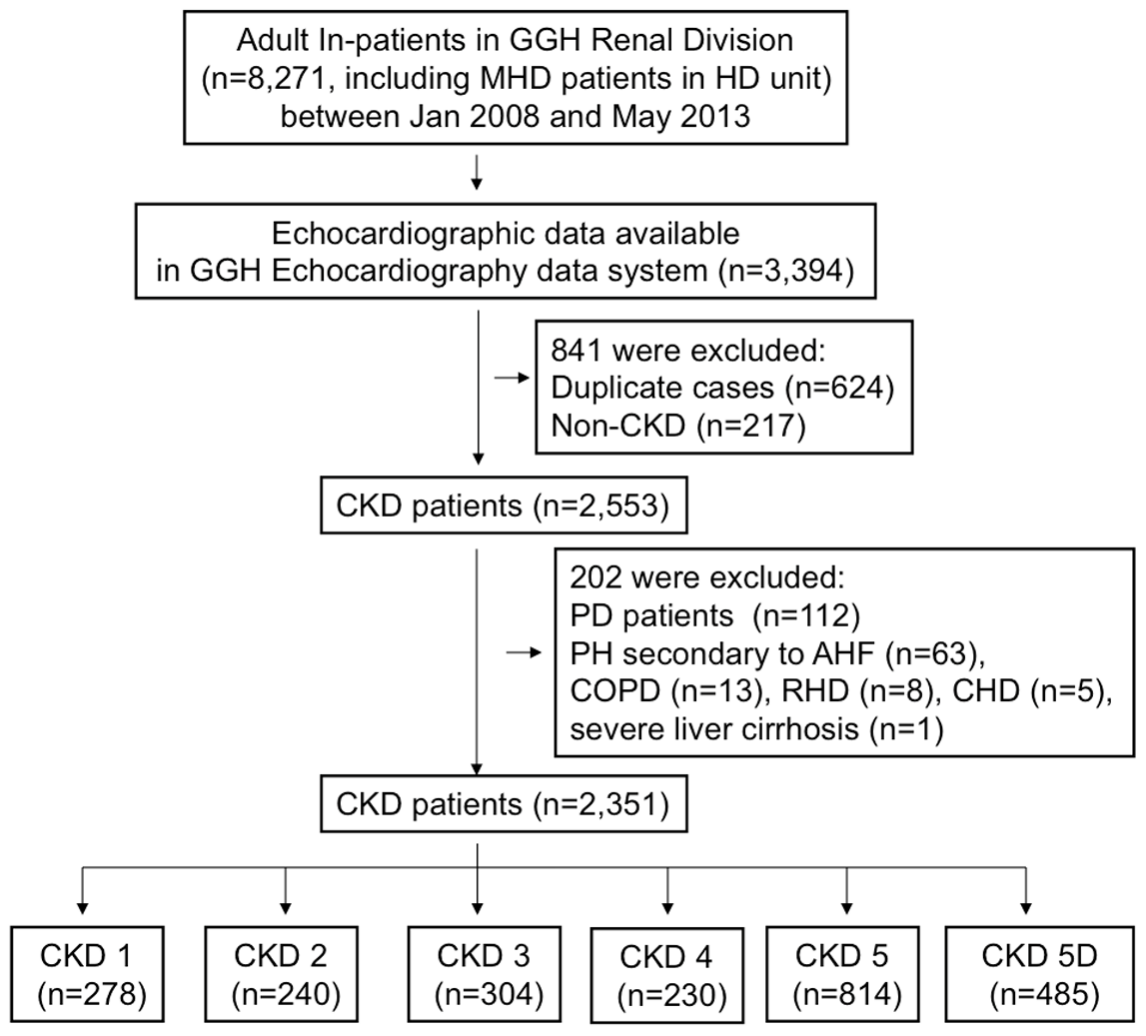
Schematic of study sample with exclusions from analysis.

Between Jan 2008 and May 2012, 3,394 adult inpatients admitted to the Renal Division were screened for eligibility by the GGH echocardiography data system. A total of 2,351 patients satisfied the inclusion and exclusion criteria (Fig. 1). All of the collected study variables were complete except for 635 patients without data regarding proteinuria (465 in the CKD-5D, 135 in the CKD-5 and 35 in the CKD-1 to CKD-4 groups).

Of the 2,351 patients, 1062 were females, and 1289 were males. The mean age was 52.5±18.0 years. Common causes of CKD were chronic glomerular diseases (42.3%), hypertension nephrosclerosis (19.2%), and diabetic nephropathy (18.2%). The demographic, clinical and biochemical parameters of the patients in different stages of CKD are summarized in Table 1.

**Table 1:** Baseline demographic and clinical parameters of different CKD stages.

Characteristic	All (n = 2351)	CKD-1 (n = 278)	CKD-2 (n = 240)	CKD-3a (n = 138)	CKD-3b (n = 166)	CKD-4 (n = 230)	CKD-5 (n = 814)	CKD-5D (n = 485)
Age (y)	52.5±18.0	34.5±15.6	46.8±17.8	54.9±17.1	51.6±16.7	55.4±17.5	54.6±16.2	60.4±14.9
Gender (male/female)	1289/1062	128/150	132/108	84/54	103/63	147/83	429/385	266/219
Systolic BP (mmHg)	149±27	128±20	140±24	148±26	145±24	154±26	158±27	150±26
Diastolic BP (mmHg)	83±16	78±13	84±15	84±17	84±14	85±15	85±16	78±17
Current/previous smoker —n (%)	568(24.2)	66(23.7)	52(21.7)	33(23.9)	35(21.1)	62(27.0)	209(25.7)	111(22.9)
Body mass index (kg/m^2^)	22.2±4.2	22.1±3.9	22.7±3.2	23.0±3.0	23.3±3.4	22.2±3.1	22.3±5.4	21.5±3.5
With diabetes —n (%)	651(27.7)	26(9.4)	43(17.9)	38(27.5)	59(35.5)	79(34.3)	241(29.6)	165(34.0)
With hypertension —n (%)	1801(76.6)	93(33.5)	140(58.3)	108(78.3)	133(80.1)	202(87.8)	754(92.6)	371(76.5)
With CAD—n (%)	264(11.2)	5(1.8)	15(5.8)	8(5.8)	15(9.0)	34(14.8)	89(10.9)	99(20.4)
eGFR (ml/min/1.73 m^2^, CKD-EPI)	11.2(5.3–51.7)	116.6±18.1	75.1±8.4	52.1±4.8	37.6±4.1	21.6±4.3	7.2±4.6	5.7±3.6
LVEF (%)	64.8±8.9	67.2±6.0	67.5±6.9	66.0±8.4	67.2±7.5	65.4±8.0	63.5±9.4	62.6±10.2
Echocardiographic findings								
Valvular disease*	365(15.5)	3(1.1)	9(3.8)	13(9.4)	10(6.0)	35(15.2)	137(16.8)	176(36.3)
Pericardial effusion	615(26.2)	38(13.7)	24(10.0)	15(10.9)	28(16.9)	57(24.8)	278(34.2)	175(36.1)
LV systolic dysfunction	159(6.8)	3(1.1)	5(2.1)	10(7.2)	5(3.0)	9(3.9)	71(8.7)	56(11.5)
LV diastolic dysfunction	731(31.1)	20(7.2)	30(12.5)	31(22.5)	166(21.1)	69(30.0)	336(41.3)	210(43.3)
PH ——n (%)	426(18.1)	6(2.2)	16(6.7)	13(9.4)	11(6.6)	35(15.2)	163(20.0)	182(37.5)
CV morbidity——n (%)	645(27.4)	12(4.3)	34(14.2)	29(21.0)	34(20.5)	60(26.1)	229(28.1)	247(50.9)
Laboratory parameters								
Proteinuria (g/24 h)	2.0(0.8–4.0)	1.7(0.6–4.4)	2.0(0.7–4.6)	1.8(0.5–4.6)	2.4(0.6–5.3)	2.5(0.8–4.2)	1.9(1.1–3.5)	NA
Hemoglobin (g/L)	103±28	129±21	127±24	124±24	114±23	102±24	84±20	99±21
Albumin (g/L)	27.3±7.8	24.5±10.7	28.9±10.1	26.4±10.0	26.5±9.5	26.6±8.2	27.6±5.6	29.9±4.6
Uric acid (µmol/L)	433±134	366±106	413±116	433±116	466±119	474±146	457±148	408±111
Cholesterol (mmo/L)	5.4±2.5	7.1±3.4	6.5±3.1	6.4±3.0	6.0±2.6	5.7±2.7	4.7±1.6	4.2±1.1
Triglycerol (mmo/L)	1.8±1.5	2.2±1.7	2.1±1.8	1.9±1.2	2.2±2.0	1.9±1.5	1.6±1.3	1.4±1.0
HDL-cholesterol (mmol/L)	1.2±0.4	1.3±0.5	1.3±0.5	1.2±0.4	1.2±0.5	1.1±0.4	1.1±0.4	1.1±0.4
LDL- cholesterol (mmol/L)	2.9±1.6	4.0±2.3	3.5±2.0	3.6±1.8	3.2±1.7	3.0±1.5	2.5±1.0	2.2±0.8

CKD: chronic kidney disease; CKD-5D: maintenance hemodialysis patients (hemodialysis≥3 months); BP: blood pressure; CAD: coronary artery disease; eGFR: evaluated glomerular filtration rate; CKD-EPI: CKD Epidemiology Collaboration; LVEF: left ventricular ejection fraction; LV: left ventricular ; PH: pulmonary hypertenstion; CV: cardiovascular; HDL: high density lipoprotein; LDL: low density lipoprotein. NA: not available; *: moderate to severe regurgitation valvular disease; Data are given as mean±SD, medians (25^th^ to 75^th^ percentiles), or numbers and percentages as appropriate.

### Epidemiology of PH and CV morbidity in different stages of CKD

**Figure 2:**
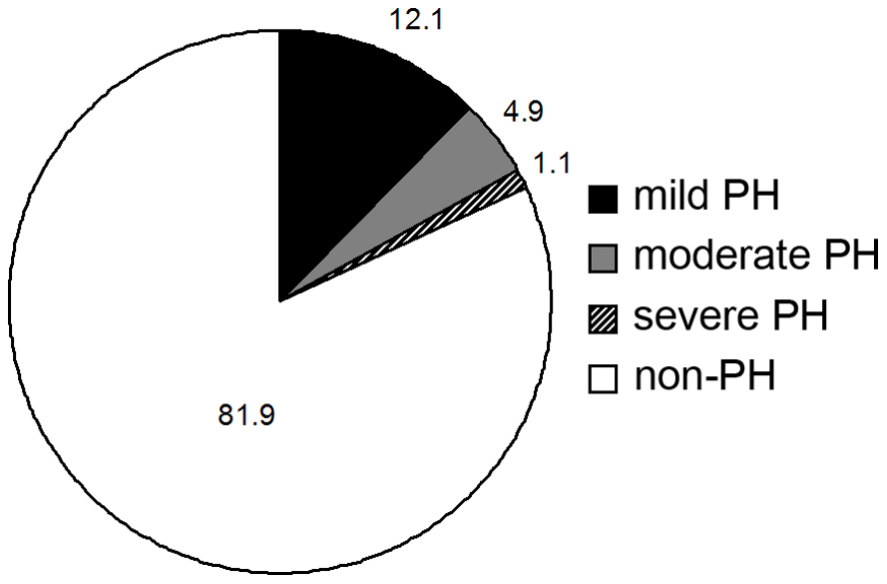
Prevalence of mild, modertate and severe PH in CKD.

**Figure 3:**
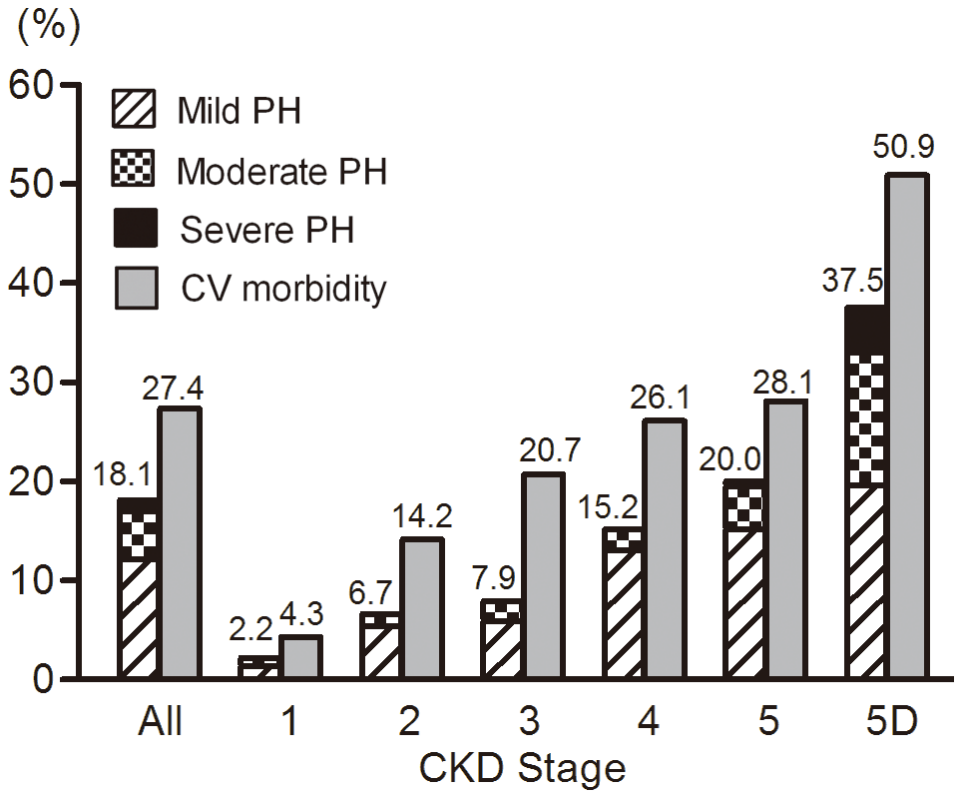
Prevalence of PH and CV morbidity in different CKD stages. Prevalence of both PH and CV morbidity increased gradually with progression of renal function, and reached a peak point when hemodialysis (CKD-5D) initiated. PH patients were mostly mild to moderate PH.

PH was detected in 426 (18.1%) patients, with a mean SPAP of 47.4±11.4 mmHg, ranging from 35 to 92 mmHg. Mild, moderate and severe PH accounted for 12.1%, 4.9% and 1.1%, respectively. In 93.9% of cases of PH in CKD, the PH was mild or moderate (Fig. 2). Severe PH was only detected in CKD Stages 5 and 5D. The prevalence of PH in CKD Stage 1-5D was 2.2%, 6.7%, 7.9%, 15.2%, 20.0% and 37.5%, respectively (Fig. 3). It was revealed that 50.5% patients with PH had pericardial effusion , 64.1% had moderate-to-severe mitral and/or aortal regurgitation, 16.9% had left ventricular systolic dysfunction, 46.2% had diastolic dysfunction, and 48.4% had CV morbidity.

**Figure 4:**
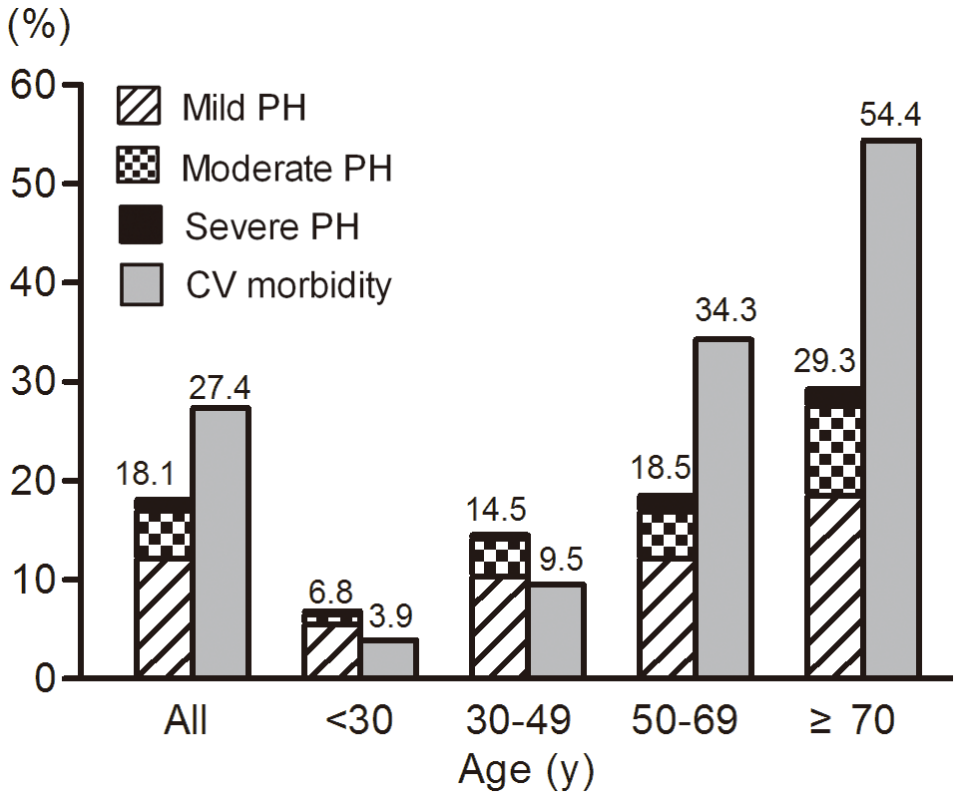
Prevalence of PH and CV morbidity stratified by different ages. Prevalence of both PH and CV morbidity showed an increasing trend with advanced ages.

**Figure 5:**
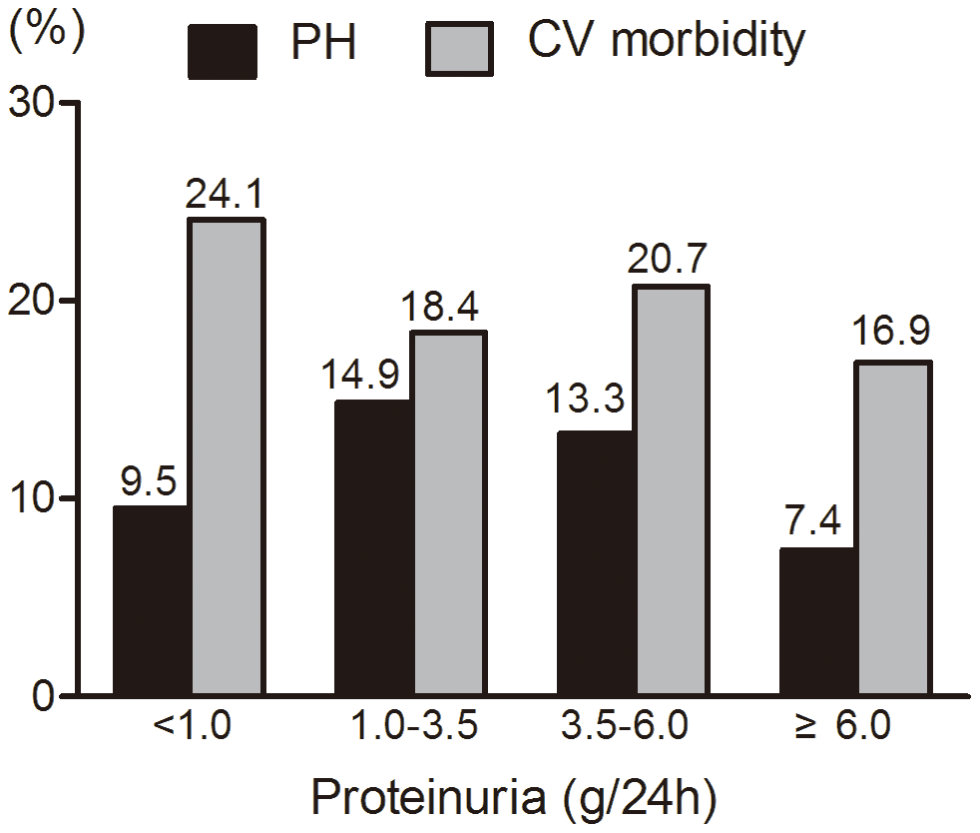
Prevalence of PH and CV morbidity stratified by proteinuria (not including CKD Stage 5D). PH was more prevalent in proteinuria ranging from 1.0 g/24 h to 6.0 g/24 h, but was less prevalent in patients with proteinuria less than 1.0 g/24 h or more than 6.0 g/24 h.

**Figure 6:**
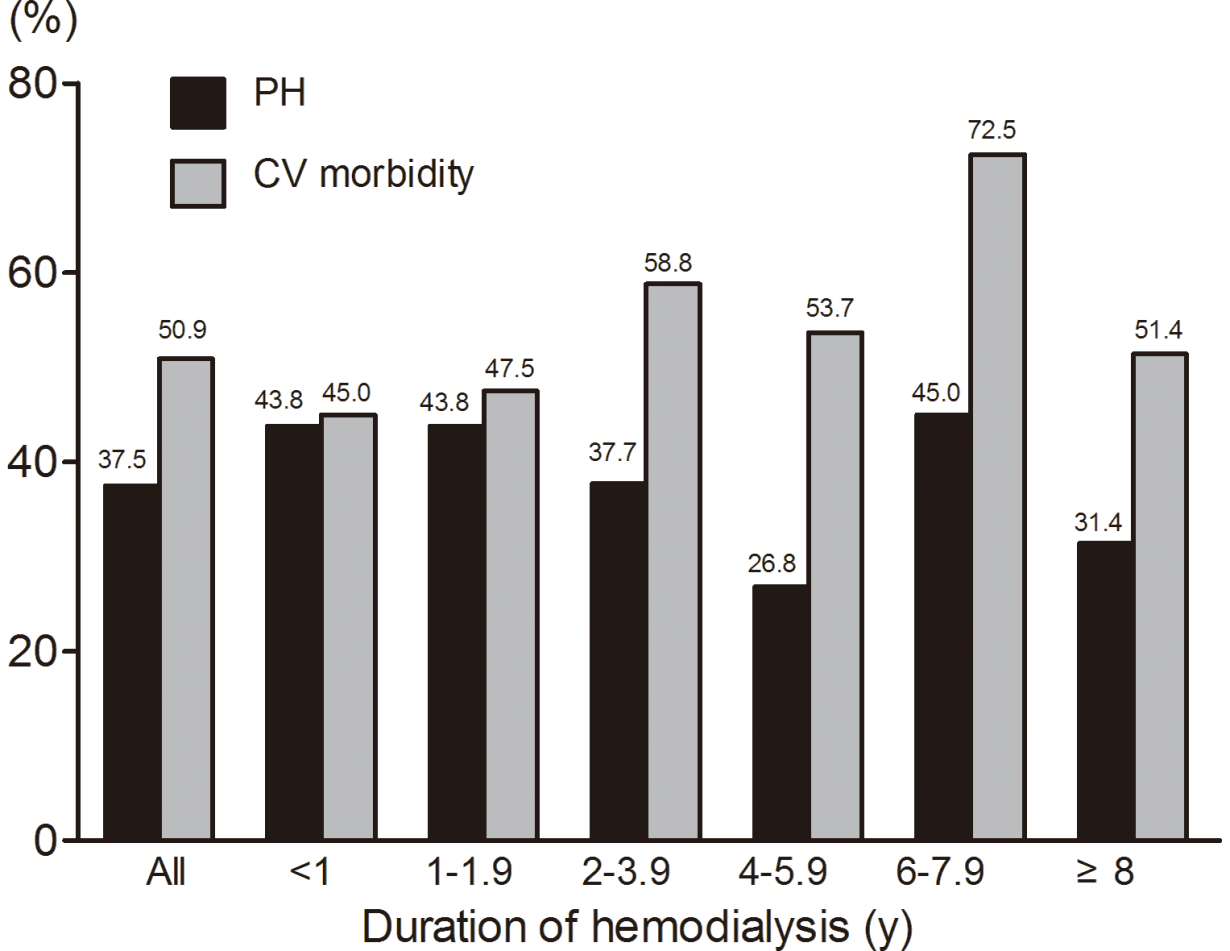
Prevalence of PH and CV morbidity stratified by duration of hemodialysis. Prevalence of neither PH nor CV morbidity showed increasing trend.

CV morbidity was found in 645 (27.4%) patients; CV morbidity in CKD1-5D was 4.3%, 14.2%, 20.3%, 26.1%, 28.1%, 50.9%, respectively (Fig. 3). After stratification by age, the prevalence of PH or CV morbidity showed an increasing trend with advanced ages (Fig. 4). When stratified by proteinuria (not including the CKD Stage 5D patients), PH was more prevalent with proteinuria ranging from 1.0 g/24 h to 6.0 g/24 h; however, PH was less prevalent in patients with proteinuria less than 1.0 g/24 h or more than 6.0 g/24 h (Fig. 5). The prevalence of both PH and CV morbidity was highest in Stage CKD-5D; however, there was not an increasing trend when stratified by the duration of HD (Fig. 6).

### Determinants of PH in CKD patients

The baseline demographic and clinical parameters of the CKD patients stratified by PH severity are shown in Table 2. Compared with the non-PH group, the patients with PH were significantly older, had lower eGFR, LVEF, hemoglobin, triglycerol, cholesterol, and LDL levels and increased incidences of diabetes, hypertension, mitral or aortal regurgitation, atrial fibrillation, pericardial effusion , cardiac dysfunction (systolic and diastolic) and previous CV events. However, there was no statistically significant difference in these variables across the three PH groups. Multivariate determinants of PH in the CKD patients are shown in Table 3. MHD, diabetes, moderate-to-severe valvular regurgitation, pericardial effusion and diastolic dysfunction were independent risk factors.

**Table 2:** Baseline demographic and clinical characteristics by degree of PH.

Characteristic	Non-PH (n = 1925)	Mild PH (n = 284)	Moderate PH (n = 116)	Severe PH (n = 26)
Age(y)	50.9±17.9	58.2±16.8^#^	61.9±15.5*^#^	63.9±11.9*^#^
Gender(male/female)	1059/866	150/134	65/51	15/11
Systolic BP(mmHg)	148±27	155±25^#^	157±25^#^	153±30
Diastolic BP(mmHg)	83±16	82±16	78±18^#^	73±17*^#^
Current/previous smoker —n (%)	466(24.2)	70(24.6)	30(25.9)	6(23.1)
Body mass index (kg/m^2^)	22.6±5.6	22.4±2.5	22.3±2.5	20.9±1.8*
Diabetes —n (%)	493 (25.6)	105(37.0)	44(37.9)^#^	9(34.6)
Hypertension —n (%)	1435 (74.5)	245(86.3)^#^	100(86.2)^#^	21(80.8)
Causes of CKD—n (%)				
Primary glomerular diseases	869(45.1)	90(31.6)	27(23.3)	9(33.3)
Hypertensive nephrosclerosis	337(17.5)	71(24.9)	34(29.3)	8(29.6)
Diabetes nephropathy	311(16.2)	74(26.0)	36(31.0)	8(29.6)
Others	408(21.1)	50(17.5)	19(16.4)	2(7.4)
eGFR(ml/min/1.73 m^2^)	16.0(5.7–62.1)	6.7(4.4–13.9)^#^	6.2(3.9±9.6)^#^	5.7(4.2–9.1)^#^
Stages of CKD—n (%)				
Stage 1	272(14.1)	4(1.4)	2(1.7)	0
Stage 2	224(11.6)	13(4.6)	3(2.6)	0
Stage 3	280(14.5)	18(6.3)	6(5.2)	0
Stage 4	195(10.1)	30(10.6)	5(4.3)	0
Stage 5	651(33.8)	124(43.7)	35(30.2)	4(15.4)
Stage 5D	303(15.7)	95(33.5)	65(56.0)	22(84.6)
Cardiac status—n (%)				
Coronary artery disease —n (%)	184(9.6)	51(17.9)^#^	26(22.4)^#^	3(11.1)
PCI or CABG	51(2.6)	14(4.9)^#^	7(6.0)^#^	0
Atrial fibrillation	20(1.0)	15(5.3)^#^	11(9.5)^#^	4(15.4)^#^
Valvular disease				
Mitral reg. mod-severe	79(4.1)	98(34.4)^#^	58(50.0)*^#^	17(63.0)*^#^
Aortal reg. mod-severe	43(2.2)	40(14.1)^#^	22(19.0)^#^	9(33.3)*^#^
Pericardial effusion (total)	400(20.7)	132(46.5)^#^	67(57.7) *^#^	16(61.5)^#^
Trace to mild	380(19.7)	128(45.1)	62(53.4)	16(61.5)
Mod-severe	20(1.0)	4(1.4)	5(4.3)	0
LV systolic dysfunction	87(4.5)	48(16.9)^#^	22(19.0)^#^	2(7.7)
LV diastolic dysfunction	534(27.7)	130(45.8)^#^	58(50.0)^#^	9(34.6)
LVEF (%)	65.8±7.8	60.4±11.5^#^	58.6±11.8^#^	61.8±9.4^#^
CV morbidity—n (%)	439(22.8)	123(43.3)^#^	67(57.8)*^#^	16(61.5)^#^
Laboratory parameters				
Proteinuria(g/24 h)	2.0(0.8–4.1)	2.3(1.3–3.9)	1.6(0.9±3.3)*	NA
Ca	2.1±0.2	2.1±0.3	2.1±0.3	2.2±0.2
Phosphorus	1.7±0.6	1.8±0.6^#^	1.8±0.6	1.8±0.6
iPTH	105(45–251)	156(77–335)^#^	180(95–337)^ #^	158(69–461)
Hemoglobin(g/L)	106±28	90±23^#^	90±23^#^	90±17^#^
Albumin(g/L)	27.2±8.2	27.1±6.3	28.3±4.6*^#^	29.3±3.5
Uric acid(µmol/L)	435±133	430±134	418±141	382±107
Cholesterol(mmo/L)	5.7±2.5	4.8±2.4^#^	4.0±1.3*^#^	3.8±0.9*^#^
Triglycerol(mmo/L)	1.9±1.5	1.5±1.7^#^	1.2±0.9*^#^	1.0±0.7^#^
HDL-cholesterol(mmol/L)	1.2±0.4	1.1±0.4	1.1±0.4	1.1±0.5
LDL- cholesterol (mmol/L)	3.0±1.6	2.5±1.5^#^	2.1±1.0*^#^	2.0±0.6^#^

CKD: chronic kidney disease; 5D: maintenance hemodialysis patients (hemodialysis≥3 months); BP: blood pressure; eGFR: evaluated glomerular filtration rate; PCI: percutaneous coronary intervention; CABG: coronary artery bypass grafting; LVEF: left ventricular ejection fraction; PH: pulmonary hypertenstion; CV: cardiovascular; HDL: high density lipoprotein; LDL: low density lipoprotein. Data are given as mean±SD, medians (25th to 75th percentiles), or numbers and percentages as appropriate. NA: not available (only 4 patients with data of 24 h-proteinuria). *: compared with mild PH group, *p*<0.05; ^#^ : compared with non-PH group, *p*<0.05.

**Table 3:** Multivariate ORs for pulmonary hypertension in CKD patients.

	Unadjusted OR(95%CI)	Adjusted OR(95%CI)	*P* value
Age(per 1 year)	1.03(1.02–1.04)		
Hemoglobin(g/L)	0.98(0.97–0.98)	0.98(0.97–0.98)	<0.001
Cholersterol(mmol/L)	0.77(0.71–0.82)		
Triglycerol(mmol/L)	0.70(0.62–0.79)		
LDL cholesterol(mmol/L)	0.68(0.61–0.75)		
Hypertension	2.08(1.56–2.79)		
Diabetes	1.71(1.37–2.14)	1.42(1.07–1.89)	0.014
CKD stage			
CKD stage 1	reference	reference	
CKD stage 2	3.24(1.25–8.41)	2.88(1.01–8.23)	0.048
CKD stage 3	3.89(1.56–9.65)	2.13(0.78–5.86)	0.142
CKD stage 4	8.14(3.36–19.72)	2.36(0.86–6.50)	0.096
CKD stage 5	11.35(4.97–25.95)	2.15(0.82–5.64)	0.120
CKD stage 5D	27.23(11.88–62.42)	5.18(2.00–13.44)	0.001
Cardiac status			
Coronary artery disease	2.19(1.64–2.91)		
Vascular disease			
Mitral reg. mod-severe	15.98(11.88–21.50)	8.14(5.81–11.40)	<0.001
Aortal reg. mod-severe	8.61(5.79–12.79)	5.77(3.51–9.49)	<0.001
Pericardial effusion	3.89(3.12–4.84)	2.28(1.73–3.00)	<0.001
LV systolic dysfunction	4.30(3.08–5.99)		
LV diastolic dysfunction	2.24(1.81–2.78)	1.32(1.01–1.73)	0.046
CV morbidity	3.17(2.55–3.94)	1.81(1.37–2.39)	<0.001

OR: odds ratio; CKD: chronic kidney disease; 5D: maintenance hemodialysis patients (hemodialysis≥3 months); LDL: low density lipoprotein ; CV: cardiovascular.

### Constitution of CV morbidity in CKD patients

Overall, 645 (27.4%) patients had CV morbidity, including 206 (48.4%) of the patients in the PH group and 439 (22.8%) of the patients in the non-PH group (*p*<0.001). The constitution of CV events is summarized in Table 4. The most common CV event was stroke, which was followed by angina pectoris and then acute heart failure. The CV events in the PH and non-PH groups had different patterns. The most prevalent CV event in the PH group was AHF, whereas in the non-PH group, the most prevalent CV event was stroke. Compared with the non-PH group, the PH group had a higher risk for cardiac disease (*p*<0.05), whereas there was no association between PH and cerebrovascular or peripheral vascular diseases (*p*>0.05).

**Table 4:** Prevalence and risks of CV morbidity in CKD patients with or without PH.

Cardiovascular events	All (n = 2351)	PH (n = 426)	Non-PH (n = 1925)	Odds Ratio (95% CI)	*P* value
All patients	645	206	439	3.17(2.55–3.94)	<0.001
All events(no. of patients with events)					
Angina pectoris	196	57	139	1.99(1.43–2.76)	<0.001
Acute myocardial infarction	76	26	50	2.44(1.50–3.96)	<0.001
Cardiac arrest	28	16	12	6.22(2.92–13.25)	<0.001
Acute heart failure	157	81	76	5.71(4.09–7.97)	<0.001
Arrhythmia for hospitalization	92	48	44	5.43(3.55–8.29)	<0.001
Stroke	265	56	209	1.24(0.91–1.70)	0.177
Transient ischemia attack	27	5	22	1.03(0.39–2.73)	1.000
Peripheral vascular disease	6	2	4	2.27(0.41–12.41)	0.298

PH was associated with cardiac morbidity but not cerebravascular nor peripheral vascular diseases. CV: cardiovascular; CKD: chronic kidney disease; PH: pulmonary hypertension.

### Association between cardiac morbidity and PH in CKD patients

A binary logistic regression analysis was performed using variables with an association of *p*≤0.1 to assess the association between cardiac diseases and PH in CKD patients. The risk of cardiac morbidity increased with PH grade; the adjusted odds ratio (OR) and 95% CI (confidence interval) were 1.79 (1.30–2.47) for mild PH, 2.75 (1.73–4.37) for moderate PH and 3.90 (1.46–10.42) for severe PH (*p*<0.001). After adjustment, there was no risk difference in CKD Stages 1–4. However, the risk of cardiac morbidity increased when the patients entered ESRD (Table 5).

**Table 5:** Multivariate ORs for cardiac morbidity in CKD patients.

	Unadjusted OR(95%CI)	Adjusted OR(95%CI)	P value
Age(per 1 year)	1.07(1.06–1.08)	1.06(1.05–1.07)	<0.001
Hemoglobin(g/L)	0.99(0.99–1.00)		
Serum albumin(g/L)	1.02(1.01–1.03)		
Cholersterol(mmol/L)	0.83(0.78–0.88)		
HDL cholesterol(mmol/L)	0.59(0.46–0.77)	0.71(0.52–0.97)	0.031
LDL cholesterol(mmol/L)	0.78(0.72–0.85)		
Hypertension	2.05(1.56–2.69)		
Diabetes	2.37(1.92–2.92)	1.42(1.11–1.82)	0.006
CKD stage			
CKD stage 1	reference	reference	
CKD stage 2	3.07(1.33–7.10)	1.51(0.61–3.76)	0.371
CKD stage 3	5.56(2.57–12.05)	1.86(0.79–4.35)	0.154
CKD stage 4	8.21(3.78–17.82)	2.32(0.98–5.48)	0.055
CKD stage 5	8.00(3.88–16.51)	2.16(0.97–4.83)	0.061
CKD stage 5D	26.21(12.69–54.15)	5.42(2.41–12.21)	<0.001
Cardiac status			
Vascular disease			
Mitral reg. mod-severe	4.01(3.06–5.27)		
Aortal reg. mod-severe	2.50(1.69–3.71)		
Pericardial effusion	1.72(1.36–2.18)		
LV systolic dysfunction	5.02(3.61–6.98)	3.95(2.62–5.96)	<0.001
LV diastolic dysfunction	2.12(1.72–2.60)		
Pulmonary hypertension			
No PH	reference	reference	
Mild PH	3.29(2.51–4.31)	1.79(1.30–2.47)	<0.001
Moderate PH	6.24(4.25–9.18)	2.75(1.73–4.37)	<0.001
Severe PH	8.70(3.91–19.36)	3.90(1.46–10.42)	0.007

Cardiac morbidity include angina pectoris, acute myocardial infarction, acute heart failure, cardiac arrest and severe arrhythmia, OR: odds ratio; CKD: chronic kidney disease; HDL: high density lipoprotein; LDL: low density lipoprotein; 5D: maintenance hemodialysis patients (hemodialysis≥3 months).

## Discussion

Earlier studies have shown that PH is present in at least 30% of CKD-5D patients. The analysis of our data, which showed the presence of PH to be 37.5% in MHD patients, was consistent with previous reports [6], [9]–[10], [31]–[32]. A major strength of this study is the large population of 2,351 patients across the spectrum of CKD who have undergone echocardiography. The overall prevalence of PH was 18.1% in the CKD population. We observed an increasing tendency towards PH with renal function decline from CKD Stage 1 to 5D, indicating that PH was not a unique phenomenon in ESRD patients, and the onset of the condition might precede dialysis treatment in many patients [3].

To the best of our knowledge, this is the first study to investigate the prevalence of PH prior to CKD Stage 4/5. PH was uncommon in CKD stage 1. It slightly increased to 6–8% in Stage 2/3 and markedly increased to 15–20% in Stage 4/5. The prevalence of PH in CKD Stage 4/5 was approximately 2 to 3-fold of that in Stage 2/3. Once hemodialysis was initiated, the prevalence of PH increased dramatically to include more than a third of the MHD patients. In comparison, the only study that reported the prevalence of PH in the CKD4/5 population based on right heart catheters was much higher (77%), with no significant difference in the prevalence between the dialysis (72%) and pre-dialysis populations [33]. However, because unexplained dyspnea was an inclusion criterion, that study does not provide a meaningful estimate of the true PH prevalence in dialysis and pre-dialysis populations [3].

Another important finding in our study was that severe PH could only be detected in ESRD patients, and 93.9% of PH cases in CKD were mild or moderate. Of the excluded patients, those in CKD Stage 1 to 4 with severe PH were found to have COPD, CHD, or RHD. This result indicated that an underlying cause should be cautiously considered if severe PH is observed in earlier stages of CKD. We did not exclude patients with PH secondary to systemic lupus erythematosus (SLE, n = 8) or ANCA-associated vasculitis (n = 6) because these two conditions are common etiologies of CKD.

The observed determinants of PH in our study could be explained by the higher prevalence of diastolic dysfunction and volume overload (resultant pulmonary congestion) in CKD patients. However, the following issues should be considered in the assessment of PH in this study. First, in this retrospective study, it was difficult to ascertain whether the echocardiograms were performed in any standardized way in terms of timing. The prevalence of PH differs before and after dialysis in CKD-5D [3]. Second, many dynamic factors could affect the estimate of the SPAP, such as the fluid status of the patients at the time of the measurement [4]. PH might occur in response to chronic volume overload. In a multicenter study that enrolled 392 hemodialysis patients, Zoccali et al. found that extravascular lung water measured by a simple, well validated ultrasound B-lines score (BL-US) was consistently associated with pulmonary pressure, left atrial volume, and ejection faction, which had prognostic value in predicting death and cardiac events [34]. In the present study, although we ruled out PH patients with AHF at the time of echocardiography, there might have been some asymptomatic PH patients with volume overload. This result could partly explain why the patients with PH suffered more from AHF. Further indirect evidence for fluid overload was the detection of pericardial effusion by echocardiography. Half of the CKD patients were found to have pericardial effusion, especially those in ESRD.

In the present study, we observed a close link between CV morbidity and PH: (1) The pattern of CV morbidity between the PH and non-PH groups differed. Patients with PH suffered more from AHF, whereas patients without PH suffered more from stroke. PH was associated with worse CV outcomes. (2) An accelerated CV risk appeared with PH patients who progressed into ESRD or who had severe PH. However, the cause and effect association between CV morbidity and PH should be further clarified. In a previous cohort study of 278 MHD patients who we followed for 2 years, we determined that PH was an independent risk factor for CV mortality and new onset events [19]. Specifically, prospective data aimed at revealing the predicative value of PH in CV outcomes in CKD patients are needed. (3) After adjustment, the predicative value of PH for cardiac morbidity exceeded that of diabetes, a well-known strong predictor of CV diseases [35]. Here proteinuria, an established risk marker for CV morbidity, was not included in our model because of its impracticability in MHD patients who usually became anuric.

Studies regarding “whether PH needs to be treated” or “how to treat PH” in CKD patients are not available. One reason for this lack of studies might be related to the “transient” SPAP in mild and moderate PH. In this retrospective study, the patients with PH had no records of any specific therapy for PH. The symptoms related to PH were not available in those patients. However, we performed a survey in 98 incident MHD patients with PH in 2011 and determined that more than 90% of MHD patients with mild PH were asymptomatic. The most common complaint in moderate to severe PH patients was fatigue, followed by peripheral edema, chest distress and dyspnea (data not shown). However, even MHD patients without PH could present with these non-specific symptoms [36]. Only one patient suffered recurrent syncope. Similarly, in Zoccali's study, among the dialysis patients with moderate-to-severe lung congestion, 71% were asymptomatic or presented with mild symptoms of heart failure [34]. This result differs from idiopathic PAH or PH secondary to COPD, scleroderma and congenital heart diseases. Therefore, echocardiography plays an important role in PH detection in asymptomatic CKD patients. Of note, patients with PH diagnosed by echocardiography without right-sided heart catheterization (RHC) should be cautious against the use of drug therapies given the multiple cardiovascular risk factors and high prevalence of diastolic dysfunction and valve regurgitation in these patients.

Our study has several potential limitations. First, the diagnosis of PH was only based on echocardiographic estimates of SPAP. No measurements were made through right-sided heart catheterization (RHC), the gold standard diagnostic tool, to confirm the echocardiographic findings. A diagnosis of PH by echocardiography might not be diagnosed as PH by cardiac catheterization. RHC could provide additional information to differentiate PH Class I and II or CKD-related PH (Class V PH). A diagnosis of PH by echocardiography without RHC could not designate the CKD patients with PH into “Class V”. So far, only two studies measured the SPAP with invasive RHC in CKD patients [18], [33]. Most studies on PH in ESRD patients used an echocardiographic estimation of SPAP for a diagnosis of PH. Second, the number of persistent PH was not available in this study. Hemodynamic changes, such as fluid status, could lead to PH. Elevated SPAP in patients with a fluid overload might be transient in cases in which the fluid status is corrected. Third, we enrolled CKD Stages 1–5 and MHD patients; we did not enroll PD patients because from 2008 through 2013, the sample size of our PD patients was small, and the enrollment of PD patients added complexity to the analysis. However, our study is notably the first and largest study group to provide epidemiological data regarding PH in all stages of CKD patients.

In summary, our study is the first report of the epidemiology profile of PH across the spectrum of CKD. PH occurs with more frequency in advanced CKD and is uncommon in Stage 1 CKD; severe PH is scarce in non-ESRD CKD. A CKD patient with severe PH by echocardiography in earlier stages should be considered to have PH from secondary causes rather than from an association with CKD. PH might be a risk factor for cardiac morbidity without being a cerebrovascular or peripheral vascular disease in CKD. Evidence of PH in predicting cardiac events from prospective studies in this population is needed.
