# Correction: Pulmonary Hypertension: Epidemiology in Different CKD Stages and Its Association with Cardiovascular Morbidity

**DOI:** 10.1371/journal.pone.0119787

**Published:** 2015-03-25

**Authors:** The PLOS ONE Staff

There are errors in the author affiliations. The affiliations should appear as shown here:

Zhilian Li^1,2^, Xinling Liang^2,1^, Shuangxin Liu^1^, Zhiming Ye^1^, Yuanhan Chen^1^, Wenjian Wang^1^, Ruizhao Li^1^, Lixia Xu^1^, Zhonglin Feng^1^, Wei Shi^1^

1 Department of Nephrology, Guangdong General Hospital, Guangdong Academy of Medical Sciences, Guangzhou, China, 510080

2 Southern Medical University, Guangzhou, China, 510515
